# Conceptualizing “Noaiphobia”: Psychophysiological stress during unaided versus AI-assisted writing in an exploratory pilot study

**DOI:** 10.1016/j.abrep.2026.100765

**Published:** 2026-10-25

**Authors:** Omar Trabelsi, Mustapha Bouchiba, Mohamed Yaakoubi, Ahmed Ghorbel, Adnene Gharbi

**Affiliations:** a The High Institute of Applied Studies in Humanities of Mahdia, Department of Education Sciences, University of Monastir, Mahdia, Tunisia; b The High Institute of Sport and Physical Education, University of Jendouba, Kef, Tunisia; c Research Unit: Physical Activity, Sport and Health, UR18JS01, National Observatory of Sport, Tunis, Tunisia; d Laboratoire Hypoxie & Poumon, UMR INSERM U1272, Université Sorbonne Paris Nord, Bobigny, France

**Keywords:** Generative AI, ChatGPT, Cognitive offloading, AI dependency, Behavioral addiction, Technostress

## Abstract

The rapid integration of generative AI into academic workflows has raised concerns about psychological dependence and about what happens when access is lost. This paper proposes “Noaiphobia” as an exploratory construct: the acute psychophysiological stress response associated with the unavailability of AI support during task execution. We tentatively frame it as arising from three processes: withdrawal-like reactions to habitual reliance, an increase in intrinsic cognitive load once an external scaffold is removed, and a threat appraisal in which task demands are perceived to exceed internal coping resources. As an initial empirical probe, an exploratory pilot study was conducted with 18 Tunisian sports science students who were daily AI users. In a within-subjects design with a fixed condition order, participants completed a short writing task in French first without AI and then with AI assistance; mean heart rate and perceived task stressfulness (visual analogue scale) were recorded in each condition. Resting heart rate had been measured in a separate session one week earlier (62.98 ± 4.78 bpm). Mean heart rate (93.10 ± 9.36 vs. 77.15 ± 5.99 bpm; MD = 15.95 bpm, 95% CI [11.56, 20.34], *p* < 0.001, *d*z = 1.81, 95% CI [1.04, 2.56]) and perceived stressfulness (73.06 ± 8.40 vs. 28.06 ± 7.44 mm; MD = 45.00 mm, 95% CI [41.53, 48.47], *p* < 0.001, *d*z = 6.44, 95% CI [4.24, 8.63]) were higher in the no-AI condition. These results offer preliminary evidence of a condition difference consistent with the proposed construct, but the fixed order and the greater effort demanded by unaided writing mean a withdrawal mechanism cannot be isolated. The study is best read as conceptual groundwork, and we outline the counterbalanced, effort-matched designs with direct measures of AI reliance needed to test the construct.

## Highlights

• Proposes “Noaiphobia” as an exploratory construct for stress when AI is absent.
• Heart rate was higher during unaided than AI-assisted writing in 18 students.
• Perceived task stressfulness was also higher in the unaided condition.
• Draws on behavioral addiction, cognitive load and transactional stress accounts.
• Exploratory pilot with fixed condition order; the mechanism remains untested.

## 1 Introduction

Generative AI applications, notably large language model (LLM) chatbots such as ChatGPT, are increasingly used by students as a writing aid, with a growing body of evidence on both the opportunities and the challenges this presents (Imran & Almusharraf, 2023). Their capabilities extend across the entire writing spectrum, offering comprehensive support for drafting initial content, refining linguistic expression, and providing robust scaffolding for complex assignments (Tarchi et al., 2025). This ready accessibility and immediate utility have fostered growing and, for some students, habitual use of these technologies in academic work (Zunaidah et al., 2023). While these tools provide immediate cognitive support by reducing the mental demands of writing (Tarchi et al., 2025), their habitual use raises critical psycho-pedagogical concerns regarding technological dependence and the corresponding potential psychological consequences of deprivation (Zunaidah et al., 2023).

According to Griffiths' “components” model of behavioral addiction, repeated and reinforcing engagement with highly rewarding activities (even in the absence of substances) can lead to the development of psychological dependence through adaptations in the brain's reward system (Griffiths, 2005). These activities are compelling because they offer immediate gratification, effortless task completion, or swift relief from negative states, thus creating a powerful reinforcement loop (Karim & Chaudhri, 2012). A central feature of this model is the presence of withdrawal symptoms when access to the rewarding stimulus is restricted or suddenly discontinued (Griffiths, 2005). These symptoms are not necessarily physical, but rather manifest as intense psychological distress and functional impairment. In the context of AI-assisted writing, frequent reliance on generative AI tools may similarly foster dependency-like patterns, such that their absence during task performance could elicit stress or anxiety responses comparable to the withdrawal component documented in contemporary measures of generative AI dependency (Goh et al., 2025).

The “cognitive load theory” (Sweller, 1988) further supports this possibility. Applying this framework, students may outsource high-element-interactivity aspects of writing to AI systems, engaging in cognitive offloading that significantly reduces intrinsic cognitive load. While this process is momentarily efficient, it limits the engagement of germane cognitive load, which is essential for the development of autonomous cognitive schemas (Jiang & Kalyuga, 2022; Leppink, 2016). Over time, this may reduce the individual's capacity to independently manage complex writing demands.

Complementing these behavioral and cognitive mechanisms, the “transactional model of stress and coping” (Lazarus & Folkman, 1984) provides a psychological framework for understanding how such dependence translates into stress. According to this model, stress arises when perceived task demands exceed available coping resources. For students who have normalized the use of generative AI as an essential cognitive support that they rely on daily and habitually, the requirement to complete complex academic writing tasks without assistance may trigger a primary appraisal of threat. This perception (that the task exceeds their unaided capabilities) can lead to a heightened psychophysiological stress response (Sun et al., 2023). This response reflects the emotional manifestation of cognitive overload (da Silva Cezar & Maçada, 2026), accompanied by reduced self-efficacy (Feldon et al., 2023) and anxiety of the kind described in the broader neurobiological literature on behavioral addiction (Choi et al., 2019).

The unavailability of AI support at the point of performance represents the critical trigger in this process. We use “deprivation” to denote any circumstance in which a habitual user must complete a task without the AI support they would ordinarily recruit, and we distinguish three contexts: (i) unanticipated interruption during the task, as when access fails through a service outage, an exhausted usage limit, or loss of connectivity; (ii) an anticipated external prohibition known before the task begins, as in proctored examinations, in-class assessments, or institutional restrictions on AI use; and (iii) voluntary abstention. “Noaiphobia” is proposed to apply to (i) and (ii), in which availability is removed by circumstance rather than by choice. In both, the external cognitive scaffold is absent precisely when the full demands of the task re-emerge, producing a rapid increase in intrinsic cognitive load that may exceed the limited capacity of working memory (van Nooijen et al., 2024) and, in survey and interview data reported by Zunaidah et al. (2023), are associated with students' own concerns about cognitive overload, difficulty functioning without the tool, and anxiety. What is abrupt is therefore the re-imposition of undelegated task demands at the moment of performance, rather than necessarily the moment at which unavailability is discovered; adaptation is precluded because the student has no opportunity to rebuild capabilities that habitual offloading has left unexercised (Chirayath et al., 2025).

At the same time, this process aligns with the concept of “technology withdrawal distress”. Earlier work on digital dependency established that interrupting access to a habitually used technology elicits discomfort, anxiety and physiological arousal, most directly in experimental studies of smartphone separation (Tams et al., 2018). Scales developed to assess generative AI dependency indicate that this form of reliance is measurable and can include a withdrawal dimension alongside cognitive preoccupation and negative consequences, though this describes what such scales measure rather than a demonstrated withdrawal response (Goh et al., 2025); users describe a progression from tool acceptance through habit formation to psychological dependence (Liu, 2026); and some users form attachment-like bonds with AI systems that exceed instrumental use (Kasturiratna & Hartanto, 2026). Generative AI differs from earlier technologies in that it substitutes for the cognitive operations constituting the task rather than for communication or entertainment surrounding it. Its removal mid-task may therefore activate withdrawal-like reactions at the point of performance.

Cognitive offloading theories provide additional support for this mechanism. Cognitive offloading refers to the delegation of cognitive processes to external tools, allowing individuals to extend their functional cognitive capacity (Risko & Gilbert, 2016). While this enhances efficiency, it may also reduce the engagement of internal cognitive resources (Jose et al., 2025). Consequently, the removal of such external support may lead to an increase in cognitive load, contributing to the anticipated stress responses.

Psychophysiological stress during academic tasks is typically reflected in measurable indicators of autonomic arousal (Roos et al., 2021). Common physiological markers include elevated heart rate (HR), increased blood pressure, and greater skin conductance, reflecting activation of the sympathetic nervous system (McEwen, 2007). At the psychological level, such activation is often accompanied by heightened anxiety, tension, or frustration (Bhoja et al., 2020). In the context of writing tasks, these indicators can capture the bodily manifestation of stress triggered by the absence of AI assistance.

Drawing on these theoretical foundations, we introduce the concept of “Noaiphobia”, defined as the acute psychophysiological stress response elicited when AI support is unavailable during an academic task that the individual would habitually complete with such support. The term is formed by analogy with “nomophobia” (no-mobile-phone phobia), the established label for the discomfort and anxiety arising when access to a habitually used mobile device is interrupted, and now the object of a substantial review literature reporting high prevalence among university students in particular (Jahrami et al., 2023). We adopt the parallel construction to signal a comparable class of technology-unavailability response. We note, however, that neither label denotes a specific phobia in the DSM-5-TR sense, and we return to this terminological question later in Section 4.2.

This phenomenon is conceptualized as arising from the interaction of three key mechanisms: (1) withdrawal-like reactions associated with habitual AI use, (2) cognitive overload following the removal of an external cognitive scaffold, and (3) the appraisal of task demands as exceeding available coping resources. Importantly, this construct emphasizes a situated, task-specific psychophysiological stress response triggered during task execution (see Fig. 1).

**Fig. 1:**
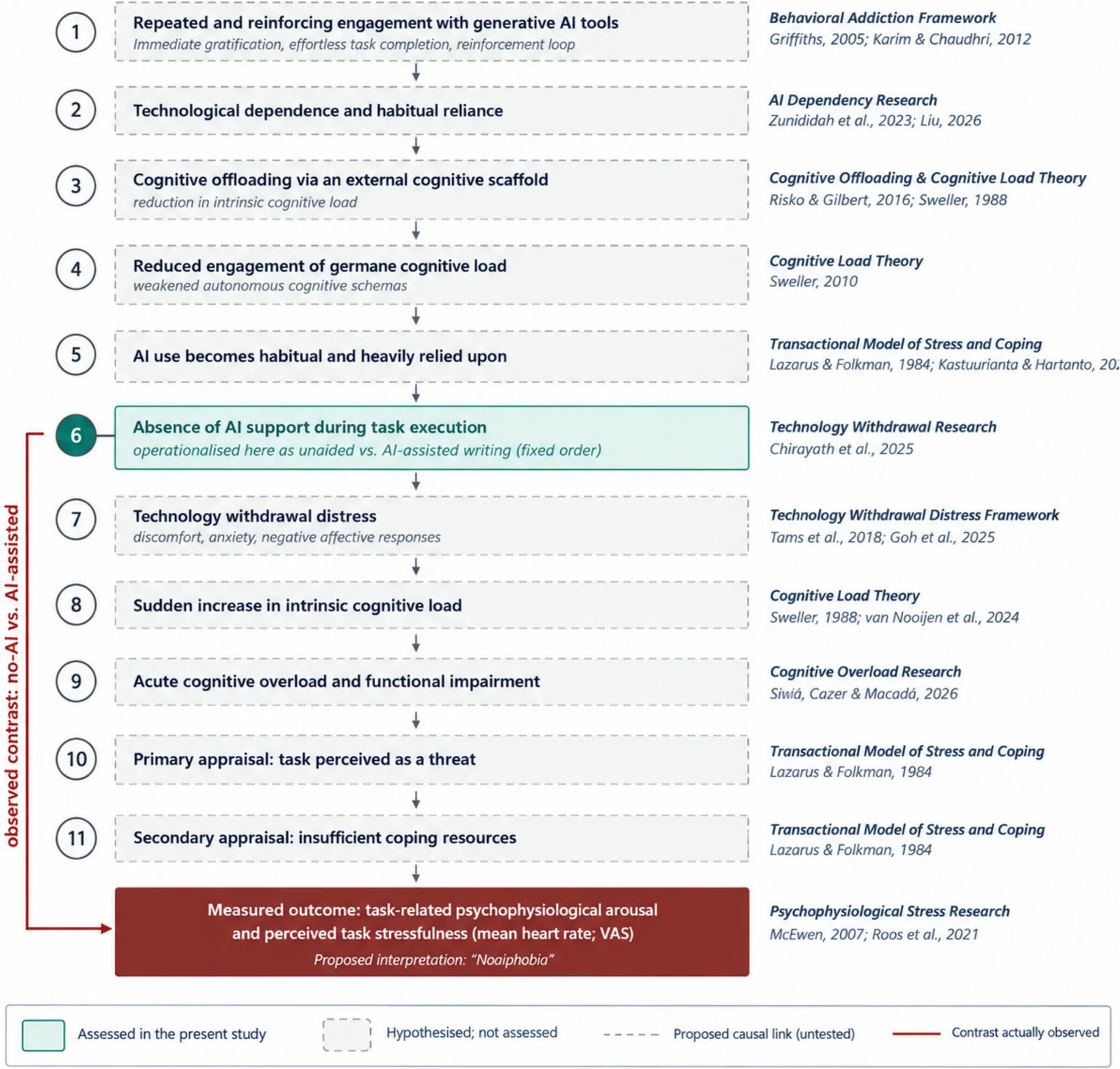
A proposed pathway to “Noaiphobia” integrating behavioral addiction, cognitive load, and transactional stress theories. Notes: (i) This sequence is a theoretical research agenda. Only the shaded stages were assessed in the present pilot study; the remaining stages and every causal link between them are hypothesized and were not tested. (ii) The three mechanisms are depicted sequentially for clarity; they may also operate as alternative or interacting explanations, which the present design cannot distinguish.

The proposed construct is related to three constructs in the emerging literature on human–AI interaction, and its position relative to each requires specification. AI anxiety refers to anticipatory apprehension about interacting with AI systems (Kim et al., 2025; Wang & Wang, 2022). It is oriented toward the presence of AI, whereas “Noaiphobia” is oriented toward its absence. The two are predicted to dissociate: individuals high in AI anxiety should show elevated distress when required to use AI, and those susceptible to “Noaiphobia” when prevented from using it.

AI dependency denotes a pattern of reliance whose validated structure comprises cognitive preoccupation, negative consequences and a withdrawal dimension (Goh et al., 2025). “Noaiphobia” is not proposed as an alternative to this construct. We position it as the acute, task-embedded expression of that withdrawal dimension: dependency measures index a general propensity reported at the trait level, whereas “Noaiphobia” denotes a state response occurring during performance of a task from which habitual support is absent. It therefore presupposes habitual reliance, though not dependency of clinical severity, and is not expected in individuals who do not habitually delegate the operations the task demands. The corresponding falsifiable prediction is that the magnitude of the response should scale with dependency scores and should be minimal or absent in non-users.

AI attachment describes the formation of emotionally significant bonds with AI agents (Kasturiratna & Hartanto, 2026). “Noaiphobia” as defined here is restricted to the loss of functional cognitive support during task performance and does not encompass relational separation from a specific agent. The two are predicted to differ in their eliciting conditions: attachment-related distress should follow separation from the agent irrespective of task context, whereas “Noaiphobia” should arise only when a task requires operations the individual habitually delegates. Whether the relational and functional pathways interact when the same system serves both roles remains an open question.

Technology withdrawal distress, finally, captures generalized responses to the absence of digital tools (Tams et al., 2018). “Noaiphobia” differs in that the stress emerges during ongoing task execution rather than during general non-use, and in that the tool removed substitutes for the cognitive operations constituting the task itself (Risko & Gilbert, 2016).

Before turning to the present study, it is worth naming directly the most obvious competing account of any such difference: unaided writing may simply be a harder task than AI-assisted writing, independent of any withdrawal or dependency mechanism. A difference in heart rate or perceived stress between a more effortful and a less effortful task is the expected consequence of task difficulty alone and does not by itself indicate a withdrawal-like process. The within-subjects comparison described in Section 2 cannot, on its own, separate a task-effort account from a withdrawal account, since the two are confounded in the primary contrast (see Sections 4.1 and 4.3). We flag this here so that the withdrawal framing developed above is read as a candidate interpretation rather than as an explanation the design has already isolated.

Given the novelty of the proposed construct, the present study aims to provide preliminary empirical evidence for the “Noaiphobia” phenomenon. Specifically, it examines whether students who habitually rely on generative AI tools exhibit increased psychophysiological stress responses when AI assistance is unavailable during a writing task. As an exploratory pilot study, it is intended to provide an initial empirical basis for the construct and inform future research. On this basis we advanced two directional hypotheses. First, mean HR would be higher when the writing task was performed without AI assistance than with it. Second, perceived task stressfulness would likewise be higher without AI assistance.

## 2 Methods

### 2.1 Participants

As summarized in Table 1, the study sample consisted of 18 sports science students (M^age^ = 21.70 ± 0.80 years). Eligibility required daily use of generative AI tools for study-related purposes (e.g., academic writing, coursework support) and a minimum grade of 75% in French language subject within their academic program. Eligibility was established by self-report during recruitment screening, in which participants confirmed daily use of generative AI tools for study-related purposes. Frequency, duration, purposes and patterns of use were not further quantified, and no measure of dependency was administered. Participants' first language was not systematically recorded at screening. French is the medium of instruction and of academic writing for this population, but for any participant whose first language is not French, the no-AI condition would additionally require second-language production without AI assistance, over and above the removal of AI-based composition support; we return to this as a limitation below.

**Table 1:** Age and Resting Heart Rate (RHR) characteristics of study sample.

Sex	*N*	Age	Mean RHR in 5 min
Males	8	21.64 ± 0.78	59.10 ± 4.24
Females	10	21.75 ± 0.85	66.08 ± 2.27
Overall sample	18	21.70 ± 0.80	62.98 ± 4.78

Sports science students were selected to ensure a relatively homogeneous sample in terms of cardiovascular condition. Admission to this program requires a mandatory medical examination confirming cardiovascular health. This was particularly important given that HR served as the primary physiological indicator of stress, as it reduced the likelihood of undetected cardiovascular conditions that could confound HR responses during task performance.

Sex was recorded by self-report during the same recruitment screening at which AI-use eligibility was established. The study was approved by the Institutional Review Board (IRB) of the High Institute of Sport and Physical Education of Kef, Tunisia (PHE-0025/2024), and was conducted in accordance with the principles of the Declaration of Helsinki. All participants provided informed consent prior to participation. Written informed consent for publication of identifiable images was obtained from each of the four participants shown in Fig. 2 at the time the photographs were taken. Faces and participant names on paperwork are redacted; where a participant's face is visible in the frame it is fully obscured by an opaque block covering the entire face, not only the eyes.

**Fig. 2:**
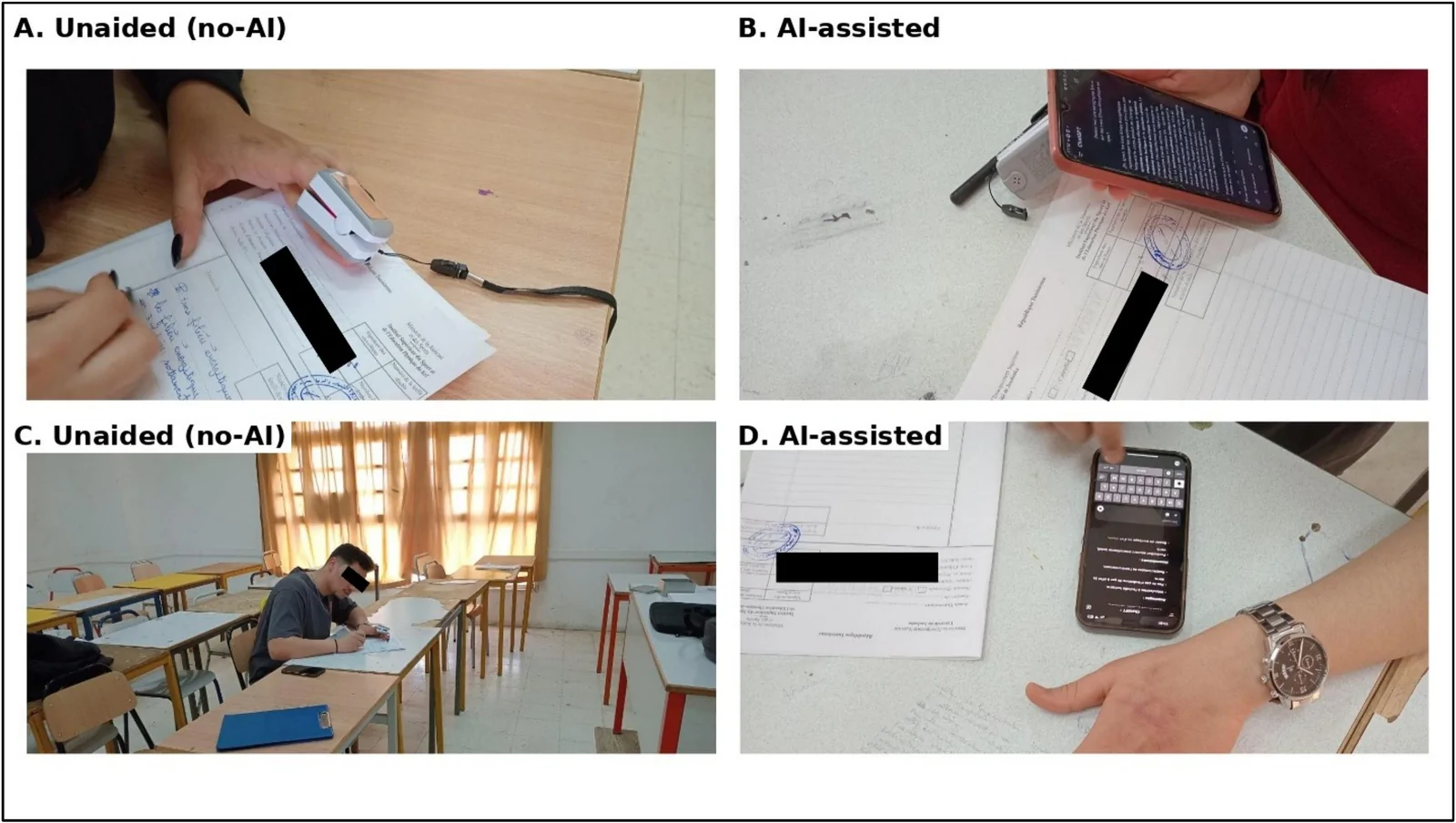
Measurement setup during actual data collection (not staged), labeled by condition (A, C: unaided; B, D: AI-assisted).

### 2.2 Procedure

Participants were tested individually in a quiet, well-lit classroom between 9:00 and 11:00 a.m. (see Fig. 2). Using a within-subjects design, they completed a 10-line writing task in French on paper, focusing on a topic familiar to sports science students (Bioenergetic systems). Familiarity with the topic was established prior to the study through a preliminary survey conducted among 245 sports science students, in which participants were asked to indicate topics they felt confident writing about. “Bioenergetic systems” emerged as the most frequently selected topic, endorsed by 78.6% of respondents. It should be noted that this 245-student sample served solely to identify a suitable writing topic and was not used to recruit or screen participants. The survey was administered as an exercise independent of the experimental recruitment described in Section 2.1, and respondents were not asked for information that would allow the two samples to be linked, so it cannot be determined whether any of the 18 experimental participants had also completed the earlier survey. The survey was conducted in the semester preceding data collection.

For the first condition, participants had 5 min to complete the task without any AI assistance. They were informed of this restriction before the task began, and smartphones were powered off and kept on the desk rather than removed from the room, since the institute's security protocol did not permit personal devices to be collected by research staff; phones were not accessed or used during the unaided trial (see Fig. 2). No AI tool was withdrawn during task execution. A 10-min rest period followed to allow psychophysiological recovery. In the second condition, participants completed the same task for 5 min with access to their habitual generative AI tools (e.g., ChatGPT, Gemini, DeepSeek) using their smartphones (see Fig. 2). Counterbalancing was not used, as starting with AI assistance would create carryover effects. Specifically, participants could generate, memorize, or internalize AI-produced content (e.g., ideas, structure, or phrasing) during the AI-assisted condition and subsequently reuse or adapt this material in the no-AI condition. This would artificially reduce the cognitive demands of the unaided task and potentially attenuate the associated stress response, thereby confounding the intended comparison between conditions.

### 2.3 Measures

#### 2.3.1 Mean heart rate (HR)

The primary outcome measure was mean HR during the 5-min writing task. HR is widely recognized as a reliable physiological marker of stress, as elevated autonomic activation, particularly sympathetic nervous system activity, leads to measurable increases in cardiac activity (increased HR) under cognitive and emotional strain (Fauquet-Alekhine et al., 2016; McEwen, 2007).

HR was recorded using a Beurer PO 80 pulse oximeter (Beurer GmbH, Ulm, Germany), a CE-marked medical device (0483CE) with a manufacturer-stated pulse-rate accuracy of ±2 bpm, per the device's instructions for use (Beurer GmbH, PO 80 pulse oximeter, instructions for use). The oximeter was worn on the index finger of the non-writing hand, continuously monitoring pulse patterns throughout each 5-min trial (see Fig. 2). Pulse rate was recorded continuously throughout each trial and processed with SpO₂ Assistant software (model CMS—SA, version 3.1.0.1), which computes mean pulse rate as the arithmetic mean of all recorded values across the recording. The software quantifies non-effective data (zero-value and manually excluded segments) as an artifact percentage; artifact was 0.0% in all recordings, and no data were excluded from any analysis. Recording began immediately before the participant started writing and ended on task completion, giving recording durations of approximately 5 to 5.2 min. After recording starts, the device screen switches off while tracking continues, reducing distraction during task completion.

In addition, a baseline (resting) HR was recorded for each participant prior to the experimental session. This measurement was obtained during a separate session conducted one week before the main experiment, under the same environmental conditions (i.e., in the same quiet, well-lit room). Participants were seated comfortably and instructed to remain at rest for 5 min while HR was continuously monitored using the same pulse oximeter device. The mean HR over this 5-min resting period was calculated and used as an individual baseline reference for subsequent comparisons.

#### 2.3.2 Perceived task stressfulness

After completing the 5-min writing task in each condition, participants rated their perceived stress using a paper-and-pencil visual analogue scale (VAS). They responded to the question in French, *“How stressful did you find this task?”* by placing an “X” on a 100-mm horizontal line. The left end of the line indicated “no stress” and the right end indicated “maximum stress”, with stress level increasing progressively toward the right anchor. VAS measures have been used for decades in research and are well-validated for assessing subjective stress (Gift, 1989; Lesage et al., 2012).

### 2.4 Data analysis

Normality of the difference scores was assessed using the Shapiro–Wilk test and by visual inspection of Q–Q plots. Because the resting measurement was obtained in a separate session one week earlier, it is reported as descriptive participant information and was not entered into the inferential analysis. Each outcome was therefore analyzed with a paired-samples *t*-test comparing the unaided and AI-assisted conditions, with significance set at *p* < 0.05. Effect sizes are reported as Cohen's *d*_z_, the mean difference divided by the standard deviation of the difference scores, which is the appropriate standardized estimator for within-subjects designs; the small-sample bias-corrected value (*g*_z_) is reported alongside. Given the sample size, Wilcoxon signed-rank tests were then conducted as nonparametric confirmation of each comparison. A sensitivity analysis indicated that with *n* = 18, α = 0.05 (two-tailed) and power of 0.80, the minimum detectable effect was *d*_z_ = 0.70. Confidence intervals for dz were obtained by inverting the noncentral t-distribution using the observed t-statistic (Cumming & Finch, 2001), which is more accurate than the normal-approximation formula at this sample size. All statistical analyses were conducted using IBM SPSS Statistics, version 29 (IBM Corp., 2022). Data are presented as mean ± standard deviation (SD) throughout, in both the text and the figures.

## 3 Results

### 3.1 Changes in mean HR

As shown in Fig. 3, mean HR was higher in the unaided condition (93.10 ± 9.36 bpm) than in the AI-assisted condition (77.15 ± 5.99 bpm). The difference was statistically significant, *t*(17) = 7.66, *p* < 0.001, MD = 15.95 bpm, 95% CI [11.56, 20.34], *d*_z_ = 1.81, 95% CI [1.04, 2.56] (*g*_z_ = 1.73). The difference scores did not deviate from normality (*W* = 0.966, *p* = 0.713), and a Wilcoxon signed-rank test confirmed the comparison (*p* < 0.001). For reference, resting HR recorded in a separate session one week earlier averaged 62.98 ± 4.78 bpm; because that measurement was obtained on a different day and under different anticipatory conditions, it is reported descriptively and is not included in the inferential comparison.

**Fig. 3:**
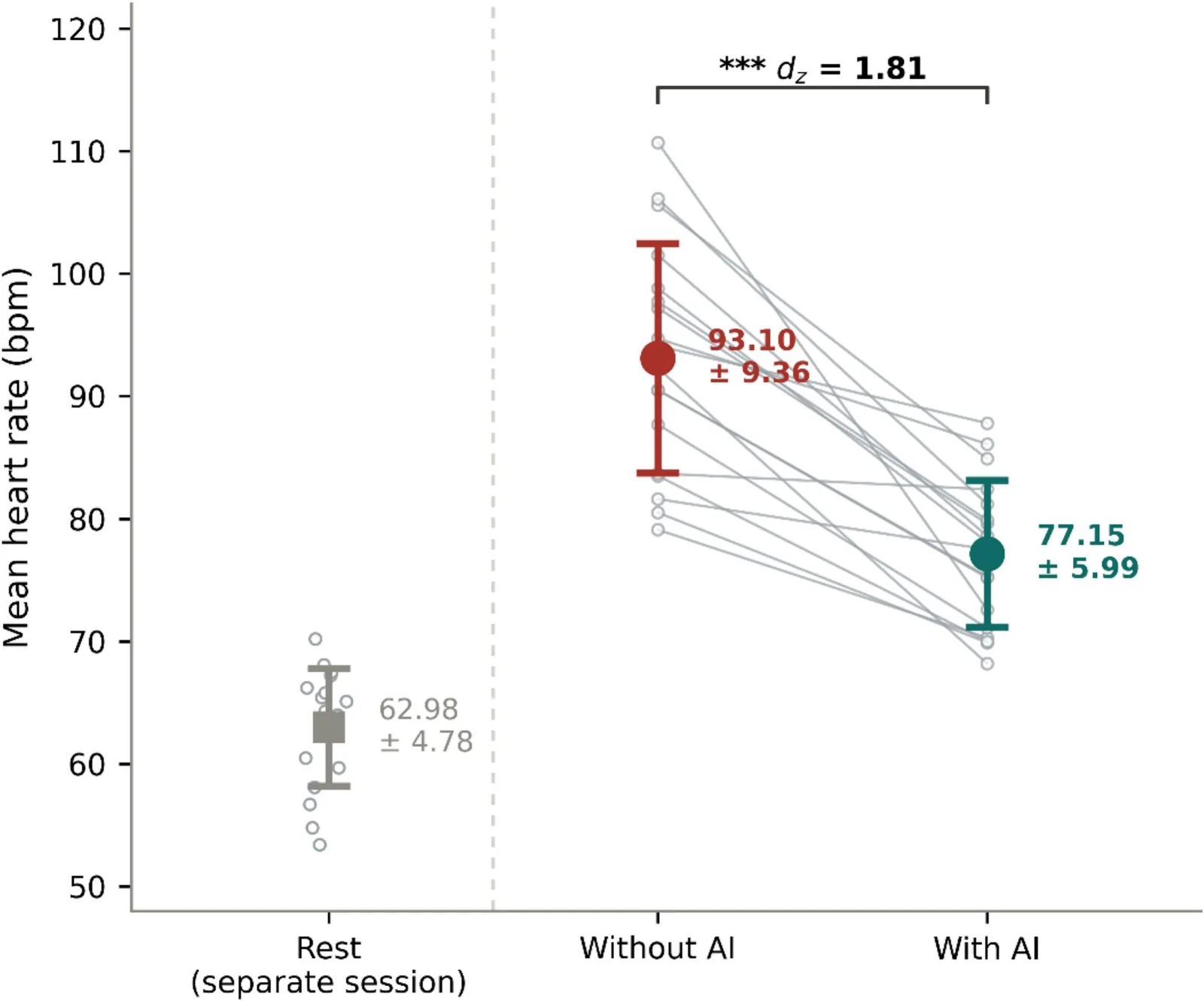
Mean heart rate in the unaided and AI-assisted conditions. Grey lines connect individual participants (*n* = 18); colored markers show condition means ± SD. Resting heart rate, shown left of the divider, was recorded in a separate session one week earlier and is presented as descriptive reference only. *** *p* < 0.001.

### 3.2 Perceived task stressfulness

As illustrated in Fig. 4, perceived task stressfulness was higher in the unaided condition (73.06 ± 8.40 mm) than in the AI-assisted condition (28.06 ± 7.44 mm), *t*(17) = 27.32, *p* < 0.001, MD = 45.00 mm, 95% CI [41.53, 48.47], *d*_z_ = 6.44, 95% CI [4.24, 8.63] (*g*_z_ = 6.15). The difference scores did not deviate from normality (*W* = 0.919, *p* = 0.124), and a Wilcoxon signed-rank test confirmed the comparison (*p* < 0.001). Changes in mean HR and changes in perceived stressfulness were positively but not significantly associated across participants (*r* = 0.44, *p* = 0.071), indicating that the two indicators moved in the same direction without being redundant.

**Fig. 4:**
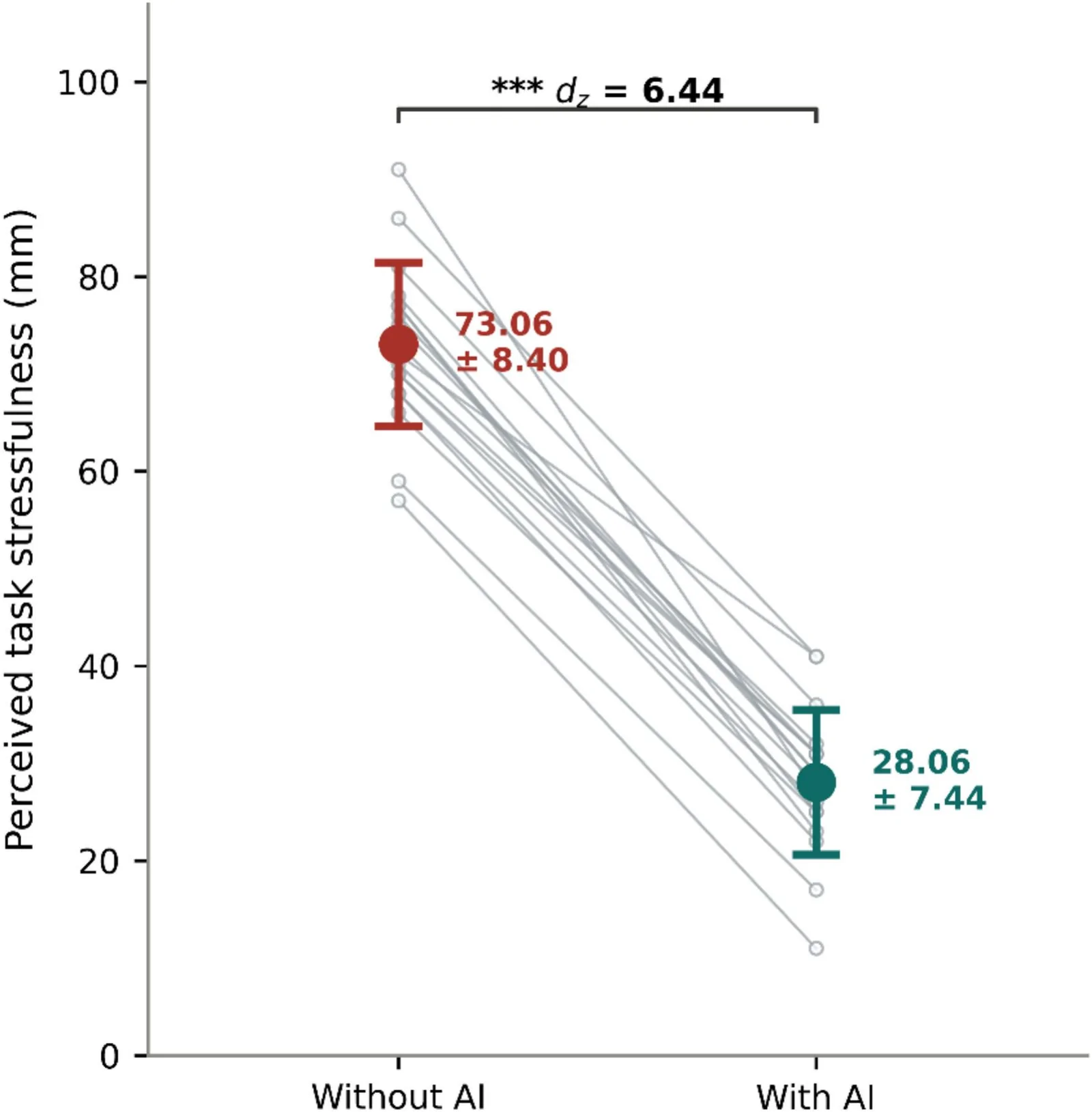
Perceived task stressfulness (VAS, mm) in the unaided and AI-assisted conditions. Grey lines connect individual participants (*n* = 18); colored markers show condition means ± SD. *** *p* < 0.001.

## 4 Discussion

This study offers preliminary evidence that, among students who use generative AI daily for writing, completing a writing task without AI assistance was accompanied by higher physiological arousal and higher perceived stress than completing the same task with assistance. Mean HR was higher in the unaided condition, and participants reported substantially higher task stressfulness. The two indicators moved in the same direction, though they were not significantly correlated across participants, suggesting that they capture partly distinct aspects of the response.

### 4.1 Interpretation of key findings

These findings extend theoretical perspectives on technological dependence. According to Griffiths' “components” model of behavioral addiction (Griffiths, 2005) repeated engagement with highly reinforcing activities (such as the high-reward, low-effort outputs provided by generative AI) can foster patterns of psychological dependence. These activities are perceived as rewarding because they offer immediate gratification and swift relief from the negative state of cognitive effort. The critical test for established dependence, according to these models, is the experience of withdrawal symptoms when access to the stimulus is restricted (Griffiths, 2005). The present design does not administer this test: habitual AI use was established by eligibility criteria rather than measured, and no withdrawal-symptom indicator was collected. With that caveat, the observed increase in psychophysiological stress (reflected in elevated mean HR and higher perceived task stressfulness in the no-AI condition) may be interpreted as a proxy for withdrawal-related distress. This pattern is consistent with, but does not test, the notion that the absence of a habitual cognitive aid can elicit responses analogous to those described in Griffiths' “components” model of behavioral addiction (Griffiths, 2005).

The present findings can also be interpreted through the lens of technology withdrawal distress, which refers to the negative psychological and physiological responses that may arise when access to frequently used digital tools is restricted (Tams et al., 2018). Scales developed to assess generative AI dependency similarly incorporate a withdrawal-type factor, although, as in Section 4.2, these sources treat dependence as an open question rather than as established prior evidence (Goh et al., 2025; Yankouskaya et al., 2025). Neither AI dependency, AI attachment nor AI anxiety was measured in the present study. It is therefore not possible to determine which form of reliance or apprehension accounts for the observed responses, and the alignment with the withdrawal literature remains interpretive. In this context, the significantly elevated HR and perceived stress observed in the no-AI condition may reflect such withdrawal-related processes. However, the phenomenon observed in the present study appears to extend beyond generalized technology deprivation. Unlike typical withdrawal contexts, the stress response emerged during active task performance, suggesting that the absence of AI not only removes a habitual tool but also removes a support that students have come to rely on for this kind of task. This combination of withdrawal-related distress and increased task demands may contribute to the magnitude of the psychophysiological response observed. Because habitual reliance was established at eligibility rather than manipulated, the withdrawal interpretation rests on participants' AI use outside the study rather than on any within-experiment removal of support. The withdrawal account is therefore one of several compatible with the data. Unaided composition is more effortful than composition with a chatbot available, and elevated arousal is the expected consequence of greater cognitive effort alone. The unaided condition may additionally have engendered frustration, concern about being evaluated, low confidence in independent writing, or difficulty retrieving the relevant content within the five-minute limit, each of which would raise HR and perceived stressfulness without invoking any withdrawal mechanism. The present design cannot adjudicate between these accounts. Doing so requires measuring dependency directly, equating task effort across conditions, and assessing frustration, writing self-efficacy and evaluative concern alongside the primary outcomes.

The application of the “Cognitive Load Theory” (Sweller, 1988) offers a candidate mechanism for the acute stress reported when generative AI support is removed. The capacity of human working memory is highly limited. Complex writing is characterized by high intrinsic cognitive load due to the intricate nature and high interdependence (element interactivity) of its components, such as synthesizing ideas, structuring arguments, and maintaining linguistic cohesion (Feldon et al., 2023). When students use AI, they are essentially performing cognitive offloading, allowing the chatbot to handle these highly interactive elements, thereby significantly reducing their intrinsic cognitive load. This repeated practice, however, prevents the engagement of germane cognitive load, which is the beneficial mental effort required to build and automate the internal cognitive schemas essential for autonomous skill mastery. Therefore, when the AI scaffold is removed, the “Cognitive Load Theory” predicts that the full demands of the complex writing task re-emerge (van Nooijen et al., 2024), imposing high-element-interactivity content that may exceed the limited capacity of working memory (Sweller, 2010). Both citations to van Nooijen et al. (2024) in this paper draw on a scoping review of expert scaffolding in visual problem-solving, not writing; we apply its account of working-memory limits to writing tasks by extension rather than as a direct finding. Cognitive load was not measured in the present study. HR and perceived stressfulness are consistent with this account but do not establish it, and elevated arousal is equally compatible with the alternative explanations considered above.

Furthermore, the Transactional Model of Stress and Coping (Lazarus & Folkman, 1984) provides the psychological framework for understanding the student's distress. This model proposes that stress is not an intrinsic property of an event but arises from a dynamic interaction (or transaction) between the individual and their environment. The process begins with primary appraisal, where the student evaluates the unaided writing task and determines it to be a threat. For a habitual AI user, their internal skills (such as structuring or synthesizing) may be less practised, consistent with the negative association between AI use and critical-thinking scores reported in correlational, offloading-mediated research (Gerlich, 2025). Therefore, the task is judged as demanding more than their current unassisted ability allows. Next is secondary appraisal, where the student assesses their coping resources (Lazarus & Folkman, 1984). Since the habitually used external resource is absent, they appraise their available internal skills as insufficient. The student perceives that the writing task demands severely outweigh their available resources. Under this model, this resource deficit appraisal is the point at which stress is predicted to arise, potentially leading to heightened psychophysiological arousal (increased HR) and the experience of negative affect (perceiving the task as stressful). On this account, the unaided task would be appraised less as a routine academic assignment and more as a demanding one, consistent with the elevated psychophysiological stress response observed, although the present design cannot confirm this appraisal process directly.

The magnitude of the stress response observed in this study may also be influenced by several contextual and individual factors. One important factor is the task domain and its associated cognitive demands. The present study employed a short writing task in a familiar academic domain to ensure baseline competence; however, tasks characterized by higher intrinsic cognitive load, such as complex argumentative writing or problem-solving in unfamiliar domains, may elicit stronger stress responses when AI assistance is unavailable (Aloia & Solomon, 2015). Conversely, tasks that are simpler or highly familiar may reduce the observed effect (Lee, 2019). These two sources are extrapolated rather than direct evidence from writing tasks: Aloia and Solomon (2015) examined physiological reactivity during interpersonal conflict rather than writing, and Lee (2019) examined task complexity in first-language speech production rather than written composition; we draw on them only for the general principle that task demand shapes stress reactivity. In addition, individual differences may play a significant role. Factors such as prior subject knowledge, writing proficiency, habitual level of AI reliance, and baseline anxiety or stress sensitivity could moderate the psychophysiological response to AI deprivation. For instance, students with stronger independent writing skills may experience less cognitive overload in the absence of AI, whereas those with greater dependence on AI tools may exhibit more pronounced stress responses (Bermeo et al., 2025). Bermeo et al. (2025) studied self-sufficiency and academic confidence in virtual learning generally, not stress responses to AI deprivation specifically; we cite it here only for the general principle that individual differences in independence from a support tool may moderate reactions to its removal.

Importantly, the framework proposes that unanticipated deprivation prevents adaptive cognitive or emotional adjustment, thereby intensifying both cognitive overload and threat appraisal processes (Chirayath et al., 2025). The present design, however, instantiates anticipated prohibition rather than unanticipated interruption. Because participants knew before beginning that AI was unavailable, they had the opportunity for anticipatory adjustment that an unexpected loss of access would preclude. If suddenness amplifies the response as the framework proposes, the values observed here may underestimate the response to genuine mid-task interruption. A related question concerns the rate rather than the timing of removal. If AI support were reduced gradually, the resulting psychophysiological response might differ. From a Cognitive Load Theory perspective, progressive withdrawal could allow for a more manageable increase in intrinsic cognitive load, enabling students to progressively re-engage their internal cognitive resources (Sweller, 2010). Similarly, the Transactional Model of Stress and Coping suggests that gradual reduction of a coping resource may allow for adaptive reappraisal and the development of alternative strategies, thereby reducing perceived threat (Biggs et al., 2017). In line with behavioral addiction frameworks, withdrawal symptoms are often less severe when disengagement occurs incrementally rather than suddenly (Griffiths, 2005). Future research should therefore examine whether graded reduction of AI assistance mitigates the stress responses observed in the present study.

### 4.2 Implications

To capture this emerging phenomenon, we introduce the term “Noaiphobia” (from “No-AI” combined with “phobia”) to denote the acute psychophysiological stress response elicited when AI support is unavailable during an academic task that the individual would habitually complete with such support.

Three clarifications about the status of the construct are warranted. First, “Noaiphobia” is proposed as a state rather than a trait: it denotes a transient response elicited by a specific situation, not a stable individual difference. Whether people differ reliably in their susceptibility to it is a separate question, and one the present design cannot address. Second, the construct is descriptive rather than clinical. It is not proposed as a diagnostic entity, and the present data speak only to arousal and perceived stress during a five-minute task, not to persistence, avoidance, or functional impairment. Third, the “-phobia” suffix is used by analogy rather than in the DSM-5-TR sense. Specific phobia in DSM-5-TR (American Psychiatric Association, 2022) requires marked, persistent and disproportionate fear of an anticipated stimulus, together with avoidance and clinically significant impairment; what we describe is situational distress during the absence of a tool, which does not meet those criteria. We retain the suffix because it follows the established usage of “nomophobia,” likewise employed descriptively rather than diagnostically and now supported by a substantial review literature (Jahrami et al., 2023).

We have nonetheless considered whether a more neutral term, such as “AI-unavailability stress” or “AI-deprivation stress”, would better match the evidence at this preliminary stage. Such phrasing describes an observation but does not name a construct: it would apply to any stress arising when AI is unavailable, including in occasional users, outside task execution, and following separation from an AI agent for relational rather than functional reasons. The phenomenon proposed here is narrower on all three counts, and a term broad enough to cover the observation is too broad to identify the construct. We therefore retain “Noaiphobia”, understood as defined above: a transient state, descriptive rather than clinical, and named by analogy with “nomophobia”. The basis for that analogy, and its supporting literature, is given above and is not repeated here.

The “Noaiphobia” construct serves as a conceptual lens for integrating and understanding the underlying risks of excessive AI dependency, as it draws on core elements from multiple theoretical frameworks. Specifically, it integrates the concepts of behavioral addiction (withdrawal distress), cognitive overload (increase in intrinsic cognitive load), and stress appraisal (perception of threat), offering a critical perspective on AI dependence. Importantly, these findings can be situated alongside prior work that has raised or called for closer scrutiny of habitual AI reliance (Yankouskaya et al., 2025; Zunaidah et al., 2023). Zunaidah et al. (2023) report that students in their sample voiced concern that habitual reliance could, in their own assessment, compromise their autonomy and resilience over time; this is a reported perception rather than a measured outcome. Yankouskaya et al. (2025), by contrast, explicitly frame the possibility of a shift from support to dependence as an open question warranting investigation rather than as an established finding; we do not treat their work as prior evidence of dependence. Our findings are consistent with the concern Zunaidah et al. raise, though they cannot establish dependence, and they do not resolve the question Yankouskaya et al. pose.

To be explicit: nothing in these findings indicates that habitual AI use constitutes a behavioral addiction, or that any participant met criteria for dependence. No measure of dependence, craving, loss of control, or functional impairment was collected, and the sample was not selected or screened for problematic use. The components model and the other frameworks drawn on in Section 4.1 are applied here as a heuristic for generating hypotheses about a novel, untested phenomenon, not as a diagnostic framework or as evidence that the processes they describe were observed.

### 4.3 Limitations

Several limitations should be acknowledged when interpreting the findings of the present study. First, the study used a within-subjects design without counterbalancing: participants always completed the unaided condition first, followed by the AI-assisted condition. This was a deliberate choice, since beginning with the AI-assisted condition would allow participants to generate, memorize, or internalize AI-produced ideas, structure or phrasing and reuse them in the unaided condition, artificially reducing task difficulty and attenuating the very difference under investigation. The consequence, however, is that AI availability is completely confounded with condition order, and this affects the primary comparison rather than only the second measurement. By the AI-assisted condition, participants had become more familiar with the task and the setting, had already retrieved relevant content and formulated an initial response, and may have been less apprehensive about being observed. Each of these would be expected to reduce arousal in the second condition irrespective of AI availability, and each therefore acts in the same direction as the hypothesized effect of AI. The observed difference should accordingly be regarded as an upper bound on the effect attributable to AI availability. The ten-minute interval between conditions was intended to allow physiological recovery and cannot separate these influences from the manipulation. Disentangling them requires counterbalancing with topics matched for difficulty, a between-subjects design, or an unaided control condition on a second topic administered after the AI-assisted condition. With the present data, order and AI availability cannot be statistically separated, and no analysis bounding the size of the order effect is possible; the upper-bound framing above is a qualitative judgment, not an estimated quantity.

Relatedly, task effort is itself a confound independent of order: composing unaided is more effortful than composing with AI assistance regardless of which condition comes first, and this alone would be expected to elevate heart rate and perceived stress without any withdrawal mechanism (see Section 4.1). This effort confound is compounded by a language factor we did not assess directly: the writing task was completed in French, the medium of academic instruction for this population, but participants' first language was not recorded at screening. If French is a second language for some participants, the no-AI condition would additionally reintroduce second-language production demands, over and above the removal of AI-based composition support, further inflating the effort difference between conditions in a way the present data cannot separate from a withdrawal account.

Second, the relatively small sample size (*n* = 18) constitutes an important constraint. The study included only sports science students who were all daily users of generative AI, resulting in a highly specific and homogeneous sample. While this homogeneity may have reduced inter-individual variability, it also limits the extent to which the findings can be generalized to broader student populations with different academic backgrounds, levels of AI use, or varying degrees of writing proficiency. Moreover, the small sample size reduces statistical power and increases the risk that the observed effects may be sample-specific, despite the large effect sizes reported. As such, the results should be interpreted with caution and viewed as preliminary. In this context, the study is best understood as a pilot investigation aimed at providing initial evidence for the proposed “Noaiphobia” phenomenon. Future research involving larger and more diverse samples is necessary to confirm the robustness, stability, and generalizability of these findings across different educational contexts.

Third, the reliance on mean HR as the sole objective indicator, together with a single-item VAS for subjective stress, captures only part of the complex psychophysiological stress response. Stress involves a broader range of physiological markers, including blood pressure and skin conductance (McEwen, 2007), which were not assessed in the present study. In addition, although perceived stress was measured using a validated single-item scale, this approach reflects only a limited dimension of the subjective experience. The use of a single-item measure was primarily motivated by time constraints within the experimental protocol, as participants were required to transition rapidly to the subsequent resting period to ensure accurate physiological recovery. Future research should adopt a more comprehensive, multimodal approach by incorporating additional physiological indicators as well as multidimensional instruments, such as the NASA Task Load Index (NASA-TLX) (Hart & Staveland, 1988), to better capture perceived workload and the full spectrum of stress responses. Relatedly, the resting value was obtained one week earlier and is confounded with day, session and anticipatory state; heart rate varies with sleep, caffeine, activity and illness, none of which was recorded, so it is reported descriptively only.

Fourth, habitual AI use was established only through an eligibility criterion of self-reported daily use. Daily use does not by itself establish heavy reliance or problematic dependency, and no validated instrument was administered to assess cognitive preoccupation, compulsive use, negative consequences, functional impairment or previous withdrawal experiences. The present data therefore cannot determine whether the observed responses reflect dependency-related withdrawal or the greater effort of unaided composition. Future studies should administer a validated measure such as the Generative AI Dependency Scale (Goh et al., 2025) and test whether dependency scores predict the magnitude of the condition difference.

Fifth, the oximeter was worn on the index finger of the non-writing hand, which was also used to operate the smartphone in the AI-assisted condition. Movement artifact may therefore have introduced measurement error in that condition specifically. Because the AI-assisted condition yielded the lower values, artifact-driven inflation would attenuate rather than produce the observed difference; systematic underestimation cannot, however, be excluded, and future work should place the sensor on a hand not involved in device operation. Relatedly, the pulse oximeter used is a CE-marked medical device, but we are aware of no independent peer-reviewed validation of its pulse-rate accuracy against electrocardiography. The accuracy specification is manufacturer-reported, and mean HR derived from photoplethysmography is not equivalent to an electrocardiographic measure.

Sixth, sex was recorded but not analyzed as a moderator: with eight male and ten female participants the study is not powered to test moderation. This matters because HR was the primary indicator and sex differences in cardiac response are well established. Descriptively, female participants showed higher resting HR (66.08 ± 2.27 vs 59.10 ± 4.24 bpm) and a larger HR difference between conditions (19.29 ± 8.86 vs 11.77 ± 7.26 bpm), whereas the difference in perceived stressfulness was almost identical (45.10 ± 8.77 vs 44.88 ± 4.42 mm). These values were not tested, but they suggest the pooled magnitudes should not be assumed to apply equally to male and female students, and that sex should be a planned moderator in adequately powered work. This applies despite the sample being selected for cardiovascular homogeneity, since that selection narrows variation within each subgroup while leaving the between-sex difference intact.

Finally, sports science students were selected because admission to the program requires a medical examination confirming cardiovascular health, which was important given that HR served as the primary physiological indicator. This population may nonetheless show altered cardiac reactivity relative to the general student body, so the absolute magnitudes reported here may not transfer to less physically trained samples.

A further set of limitations concerns verification that was not built into the protocol. No manipulation check was administered: the AI-assisted condition relied on participants' own smartphones and habitual tools, and no log of time spent on the device, screenshot of the interaction, or post-task item confirmed that AI was in fact used, or how. Recovery between conditions was assumed rather than verified: heart rate was not recorded at the end of the ten-minute rest interval, so we cannot confirm that it had returned to within a defined margin of the resting baseline before the second condition began. Standard physiological covariates that can influence heart rate, including caffeine and nicotine intake, sleep, prior exercise, and, for female participants, menstrual cycle phase, were not recorded, which is a particular concern for a sample tested in the morning and selected for sports-science training. Finally, participants were not blind to the study's purpose, the two conditions differed in an obviously salient way, and perceived stress was collected as a single retrospective item immediately after each condition; demand characteristics cannot be ruled out. Future work should incorporate a manipulation check, monitor or statistically control relevant covariates, verify physiological recovery before each condition, and consider embedding the stress item among filler items with a suspicion probe.

A related limitation is that no independent measure of cognitive load or writing performance was obtained. The increase in intrinsic cognitive load proposed in Section 4.1 is inferred entirely from heart rate and perceived stressfulness; the written products themselves, though retained on paper, were not scored for word count, completion, or quality in either condition. Such scoring would have provided a first direct test of the cognitive-load account at negligible additional cost, and we identify it as a priority both for reanalysis of the existing materials and for the design of future studies.

## 5 Conclusions

The present pilot study offers initial observations suggesting that for students who have integrated generative AI into their daily academic workflows, completing a writing task without AI assistance was associated with heightened psychophysiological strain relative to completing it with assistance. This is primarily reflected in the significantly elevated mean HR recorded during the unaided writing condition, which was found to be higher than in the AI-assisted condition, indicating a measurable increase in autonomic arousal. These physiological trends align closely with participants' subjective reports, which characterized the absence of AI assistance as significantly more stressful than the supported task. The convergence of these objective and subjective markers offers preliminary evidence of a condition difference consistent with the proposed construct of “Noaiphobia”, conceptualized here as a situated, performance-based stress response triggered by the removal of an established AI scaffold. From a theoretical perspective, this pattern is compatible with, but does not establish, the multi-stage framework proposed earlier: it could reflect some combination of withdrawal-like reactions to habitual technology reliance, an increase in intrinsic cognitive load as the task's full demands re-emerge, and a threat appraisal in which students perceive their resources as insufficient. As discussed in Section 4.1, the present design cannot adjudicate between these accounts or rule out simpler explanations such as the added effort of unaided writing.

However, given the exploratory nature of this investigation and the specific, homogeneous sample involved, these findings should be interpreted with caution and viewed as provisional rather than definitive. Rather than establishing conclusive proof, this work serves as a preliminary pilot intended to lay the conceptual and empirical groundwork for future research on the stress responses associated with the unavailability of habitually used AI tools. Future research utilizing larger, more diverse populations and comprehensive, multimodal measurement approaches (including a wider array of physiological markers and multidimensional workload scales) will be essential to further validate, refine, and extend the conceptualization of the “Noaiphobia” phenomenon.

## Declaration of generative AI use

The authors confirm that no AI tools were used to generate the scientific content, analyses, or data presented in this manuscript. The tool Paperpal was used exclusively to enhance the readability and language quality of the text. All revisions were subsequently reviewed and approved by the authors, who take full responsibility for the content of the manuscript.

## CRediT authorship contribution statement

**Omar Trabelsi:** Writing – original draft, Conceptualization, Methodology, Investigation. **Mustapha Bouchiba:** Writing – review & editing, Formal analysis. **Mohamed Yaakoubi:** Writing – original draft, Visualization, Investigation. **Ahmed Ghorbel:** Writing – review & editing, Software, Data curation, Visualization. **Adnene Gharbi:** Project administration, Writing – review & editing, Resources.

## Patient consent statement

Informed written consent was obtained from all individual participants included in the study.

## Ethics approval statement

The study protocol, including the consent procedures, was reviewed and approved by the Institutional Review Board (IRB) of the High Institute of Sport and Physical Education of Kef, Tunisia (Protocol Number: PHE-0025/2024). The study was conducted in accordance with the ethical principles outlined in the Declaration of Helsinki.

## Code availability

Analyses were conducted in SPSS v29; no custom code was written.

## Funding statement

This research received no specific grant from any funding agency, commercial, or non-profit sectors. The study was conducted using institutional resources.

## Declaration of competing interest

The authors declare that they have no known competing financial interests or personal relationships that could have appeared to influence the work reported in this paper.

## Data Availability

Data will be made available on request.
